# Selections

**Published:** 1897-08

**Authors:** 


### Selections.

### A DANGEROUS POPULAR ANTISEPTIC.

Ledermann publishes in the *New York Medical Journal* for May 18 an account of a case of diffuse external otitis, of an extremely severe and resistant type, due to taking a friend's advice and treating a slight itching of the ears with carbolic acid. The strength employed is not known: "some pure carbolic acid was poured into a glass of water and syringed into the ears." Ocular symptoms and carboluria showed the systemic poisoning, and the patient was in great danger of atresia of one of her auditory canals.

The popular knowledge that carbolic acid is an antiseptic is productive of a great deal of harm. Surgeons have until recently regarded it as indispensable to have their instruments in a tray of carbolic solution, which, if strong enough to sterilize the instruments, destroyed the surgeon's hands for practical purposes; and if, as was usually the case, it was so weak as not to actually burn the hands, did no good as an antiseptic,—circumstances which were bad for the patient, the surgeon, and all concerned. The disadvantages of carbolic acid, however, can be best seen in out-patient clinics, whither patients continually come with the skin of their hands parboiled and peeling off, exposing the raw subcutaneous tissue more or less eroded by the carbolic acid which a kind friend had advised him to use for a slight cut, or burn, or abrasion. If the lay mind could only be made to appreciate that carbolic acid is always dangerous and seldom efficient as an antiseptic, a great deal of unnecessary suffering would be prevented.—*Boston Medical and Surgical Journal.*

### LYSOL TO REPLACE CARBOLIC ACID.

"In the early years of the employment of carbolic solution, the daily papers kept a permanent heading for cases of carbolic acid poisoning. It cannot be denied that the introduction of lysol solutions has resulted in a marked improvement."

This editorial utterance of the *Boston Medical and Surgical Journal* tersely confirms the well-substantiated fact that lysol is an effective substitute for phenol or carbolic acid, and at the same time is practically harmless. It takes eight parts of lysol to equal one part of carbolic acid in toxic effect, and whereas a dose of carbolic acid, taken intentionally or by accident, is usually fatal, with frightful effect and suffering, a toxic dose of lysol would not cause the same awful symptoms, and also affords much better, almost sure, chances of recovery under proper treatment. There is on record a case of a four-year-old boy drinking one ounce of lysol; four hours later the first steps at treatment were taken, the boy's stomach was washed repeatedly, and liberal doses of milk then administered; the next morning the boy had fully recovered.

The suggestion from this brief statement of facts is patent carbolic acid is a dangerous agent, and the danger is aggravated by the universal use to which it is put and the consequent familiarity of the public with it; having a perfect substitute in lysol five times more effective and one-eighth as toxic as carbolic acid, it is the manifest duty of physicians and pharmacists to give lysol the preference, and to prescribe and recommend it wherever available.—*Notes on New Remedies.*

---

### ORIGIN OF GIANT CELLS.

Anton Brosch (*Virchow's Archives*, 1896) concludes as follows:

1. Giant cells may not only originate from degenerated angioblasts, endothelia, white blood-corpuscles, etc., but also, in certain instances, from newly formed vessels of large calibre, as a result of a peculiar (perhaps tuberculous) affection of the vessel wall and a coexistent, still unknown, regressive metamorphosis. The existence of large giant cells with double nuclear wreath may be taken as proof of the accuracy of this assumption.

2. It is also possible that obliteration of the vessel lumen by proliferation of diseased intima cells (endothelial cells), and the formation of ectases by such obliteration, or by compression and distortion from without (by cellular infiltration and nodule formation in the immediate neighborhood), may play an important *rôle* in favoring the generation of giant cells from newly formed vessels of large calibre.

3. Since connective tissue occurs everywhere in the organism, and, as Bizzozero and Bozzolo have shown, can assume an endothelioid character, it is not improbable that all giant cells are derivatives of endothelium, or of endothelioid connective-tissue cells.—*Journal of Cutaneous and Genito-Urinary Diseases.*

